# Supplementary material for: Multi‐dataset identification of innovative feature genes and molecular mechanisms in keratoconus
Source: J Cell Mol Med. 2024 Sep 19;28(18):e70079. doi: 10.1111/jcmm.70079 (PMC11412914; doi:10.1111/jcmm.70079)
Supplement: Supplementary file 2 — Material S2. [file JCMM-28-e70079-s002.zip › 7.GSEA analysis/4.MYRF/KEGG_STEROID_HORMONE_BIOSYNTHESIS.html]

Details for gene set KEGG\_STEROID\_HORMONE\_BIOSYNTHESIS[GSEA]

|  || Dataset | MYRF.group.cls#h\_versus\_l |
| Phenotype | group.cls#h\_versus\_l |
| Upregulated in class | l |
| GeneSet | KEGG\_STEROID\_HORMONE\_BIOSYNTHESIS |
| Enrichment Score (ES) | -0.4518388 |
| Normalized Enrichment Score (NES) | -1.3751779 |
| Nominal p-value | 0.032520324 |
| FDR q-value | 0.25714412 |
| FWER p-Value | 0.903 |
Table: GSEA Results Summary

  

Fig 1: Enrichment plot: KEGG\_STEROID\_HORMONE\_BIOSYNTHESIS      
 Profile of the Running ES Score & Positions of GeneSet Members on the Rank Ordered List

  

| SYMBOL | TITLE | RANK IN GENE LIST | RANK METRIC SCORE | RUNNING ES | CORE ENRICHMENT || 1 | UGT1A8 | na | 369 | 0.620 | 0.0538 | No |
| 2 | UGT1A6 | na | 2556 | 0.320 | 0.0240 | No |
| 3 | UGT2B4 | na | 3822 | 0.260 | 0.0145 | No |
| 4 | HSD3B1 | na | 3864 | 0.259 | 0.0403 | No |
| 5 | CYP17A1 | na | 6124 | 0.188 | -0.0053 | No |
| 6 | CYP3A4 | na | 6458 | 0.179 | 0.0037 | No |
| 7 | UGT2B11 | na | 6521 | 0.178 | 0.0204 | No |
| 8 | UGT2B28 | na | 7657 | 0.147 | 0.0029 | No |
| 9 | SRD5A2 | na | 8005 | 0.138 | 0.0073 | No |
| 10 | STS | na | 8288 | 0.132 | 0.0129 | No |
| 11 | HSD17B12 | na | 8309 | 0.131 | 0.0259 | No |
| 12 | CYP11A1 | na | 8417 | 0.129 | 0.0362 | No |
| 13 | SULT2B1 | na | 8876 | 0.119 | 0.0354 | No |
| 14 | CYP11B2 | na | 9857 | 0.098 | 0.0173 | No |
| 15 | UGT2B10 | na | 12166 | 0.061 | -0.0429 | No |
| 16 | HSD17B1 | na | 12282 | 0.059 | -0.0400 | No |
| 17 | SRD5A1 | na | 13946 | 0.039 | -0.0839 | No |
| 18 | CYP11B1 | na | 14536 | 0.032 | -0.0976 | No |
| 19 | COMT | na | 15993 | 0.018 | -0.1377 | No |
| 20 | UGT2A3 | na | 16075 | 0.017 | -0.1383 | No |
| 21 | UGT2B7 | na | 16473 | 0.013 | -0.1484 | No |
| 22 | HSD17B8 | na | 17472 | 0.004 | -0.1767 | No |
| 23 | HSD3B2 | na | 18761 | -0.008 | -0.2130 | No |
| 24 | HSD17B7 | na | 20444 | -0.027 | -0.2587 | No |
| 25 | CYP21A2 | na | 20479 | -0.027 | -0.2569 | No |
| 26 | SRD5A3 | na | 22575 | -0.053 | -0.3117 | No |
| 27 | UGT2B15 | na | 26025 | -0.113 | -0.3994 | No |
| 28 | CYP7B1 | na | 26965 | -0.134 | -0.4125 | No |
| 29 | CYP7A1 | na | 27404 | -0.145 | -0.4101 | No |
| 30 | AKR1D1 | na | 28385 | -0.172 | -0.4204 | No |
| 31 | HSD17B3 | na | 29475 | -0.205 | -0.4305 | Yes |
| 32 | UGT2A1 | na | 29490 | -0.205 | -0.4096 | Yes |
| 33 | CYP3A43 | na | 30562 | -0.244 | -0.4151 | Yes |
| 34 | HSD17B6 | na | 31574 | -0.294 | -0.4137 | Yes |
| 35 | HSD17B2 | na | 31597 | -0.295 | -0.3837 | Yes |
| 36 | AKR1C3 | na | 31881 | -0.311 | -0.3595 | Yes |
| 37 | CYP19A1 | na | 32433 | -0.345 | -0.3395 | Yes |
| 38 | CYP3A7 | na | 32486 | -0.350 | -0.3047 | Yes |
| 39 | HSD11B2 | na | 32661 | -0.362 | -0.2721 | Yes |
| 40 | HSD11B1 | na | 32861 | -0.377 | -0.2386 | Yes |
| 41 | AKR1C4 | na | 33159 | -0.403 | -0.2054 | Yes |
| 42 | SULT1E1 | na | 33389 | -0.430 | -0.1673 | Yes |
| 43 | AKR1C1 | na | 33588 | -0.454 | -0.1258 | Yes |
| 44 | CYP1A1 | na | 33636 | -0.460 | -0.0794 | Yes |
| 45 | CYP1B1 | na | 33990 | -0.521 | -0.0354 | Yes |
| 46 | CYP3A5 | na | 34113 | -0.546 | 0.0177 | Yes |
Table: GSEA details [plain text format]

  

Fig 2: KEGG\_STEROID\_HORMONE\_BIOSYNTHESIS      
 Blue-Pink O' Gram in the Space of the Analyzed GeneSet

  

Fig 3: KEGG\_STEROID\_HORMONE\_BIOSYNTHESIS: Random ES distribution      
 Gene set null distribution of ES for **KEGG\_STEROID\_HORMONE\_BIOSYNTHESIS**
